# A Simple HPLC Method for the Quantitative Determination of Silybin in Rat Plasma: Application to a Comparative Pharmacokinetic Study on Commercial Silymarin Products

**DOI:** 10.3390/molecules24112180

**Published:** 2019-06-10

**Authors:** Eun-Sol Ha, Dong-Gyun Han, Seong-Wook Seo, Ji-Min Kim, Seon-Kwang Lee, Woo-Yong Sim, In-Soo Yoon, Min-Soo Kim

**Affiliations:** Department of Manufacturing Pharmacy, College of Pharmacy, Pusan National University, Busan 46241, Korea; edel@pusan.ac.kr (E.-S.H.); hann9607@pusan.ac.kr (D.-G.H.); sswook@pusan.ac.kr (S.-W.S.); jiminkim@pusan.ac.kr (J.-M.K.); lsk7079@pusan.ac.kr (S.-K.L.); popo923@pusan.ac.kr (W.-Y.S.)

**Keywords:** silybin, HPLC, silymarin product, rat, comparative pharmacokinetics

## Abstract

Silybin (SBN) is a major active constituent of silymarin, a mixture of flavonoids found in fruits and seeds of milk thistle. The aim of this study was to describe a simple bioanalytical method for quantifying SBN in rat plasma. A simple protein deproteinization procedure with acetonitrile (ACN) was employed for plasma sample preparation. A reversed column and gradient elution of a mobile phase (mixture of phosphate buffer (pH 5.0) and ACN) were used for chromatographic separation. The selectivity, linearity (50–5000 ng/mL), precision, accuracy, recovery, matrix effect, and stability for this method were validated as per the current Food and Drug Administration (FDA) guidelines. Our method for SBN was applied to a comparative pharmacokinetic study on four different commercial silymarin products. This in vivo rat study demonstrated that product #4 significantly enhanced the relative oral bioavailability of SBN, as compared to product #1–3. Therefore, the bioanalytical method proposed herein could serve as a promising alternative for preclinical pharmacokinetic studies on silymarin products and, by extension, clinical use after partial modification and validation.

## 1. Introduction

**Figure 1:**
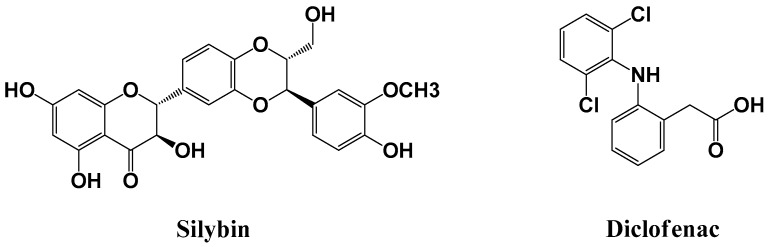
Chemical structures of silybin (SBN) and diclofenac (internal standard, IS).

Milk thistle (*Silybum marianum* L.) is a well-recognized medicinal plant widely used to prevent and treat various acute and chronic liver disorders [1]. Silymarin is a mixture of flavonoids found in fruits and seeds of milk thistle [2,3]. It is known as a clinically effective hepatoprotectant against alcoholic liver disease, toxin-/drug-induced hepatitis, viral hepatitis, and cirrhosis [4]. Silybin (SBN; Figure 1), a flavone, is the major and most active constituent of silymarin, constituting approximately 60%–70% [5]. It possesses antioxidant, anti-inflammatory, antifibrotic, hepatoprotective, and anticancer activities [6]. However, the oral bioavailability of SBN is very poor (0.73% in rats) due to its low aqueous solubility and permeability, limiting its wide clinical application [5,7]. Thus, the determination of relevant plasma pharmacokinetic parameters (including AUC, C_max_, and bioavailability) would be essential in developing new silymarin formulations to enhance the oral absorption of SBN, the major active compound of silymarin [4,8,9].

So far, several bioanalytical methods using high-performance liquid chromatography (HPLC) combined with ultraviolet/visible (UV) [5,10,11,12] and mass [13] detectors have been reported for the quantitative determination of SBN in human and rat plasma samples. However, these methods have some limitations and need for improvements on such aspects of insufficient sensitivity with a lower limit of quantification (LLOQ) of 0.5 μg/mL [5,10], time-consuming procedures of sample preparation with liquid–liquid extraction [11,12], and relatively expensive instrumentation for mass spectrometry [13]. Thus, there is a need to develop an alternative bioanalytical method with simpler sample preparation procedure and sufficient sensitivity for pharmacokinetic research and development.

Here, we developed and fully validated a simple and efficient HPLC method for quantifying SBN in rat plasma. The linearity, sensitivity, precision, accuracy, recovery, matrix effect, and stability of this HPLC method were assessed [14,15]. Next, the developed bioanalytical method was applied to investigate the comparative pharmacokinetics of SBN following the oral administration of commercially available functional food products of silymarin that were manufactured by four different pharmaceutical companies located in South Korea.

## 2. Results and Discussion

### 2.1. Method Development

Several conditions relevant to chromatographic analysis were assessed for acceptable sensitivity and good separation of the analytes from the endogenous matrix substances and metabolites within a suitable run time. Several trials were performed to select a suitable stationary phase, internal standard (IS), and sample preparation procedures.

To choose a stationary phase, several types of HPLC columns including Kinetex^®^ C8 and C18 columns (250 × 4.6 mm, 5 μm, 100 Å; Phenomenex) and Luna^®^ HILIC column (150 × 3 mm, 5 μm, 200 Å; Phenomenex) were evaluated. Our test revealed that Kinetex^®^ C18 column exhibited better resolution and intensity of peaks compared to other columns (data not shown). Thus, Kinetex^®^ C18 column was chosen as the stationary phase for SBN.

Several compounds such as celecoxib, repaglinide, ketoconazole, and quinidine were tested as potential IS that could compensate for possible analytical errors. However, these were found to be unsuitable owing to poor separation from SBN and endogenous plasma components. We finally settled for diclofenac (Figure 1) as it exhibited good separation; additionally, it displayed acceptable peak resolution, retention time, and UV absorbance intensity at the same wavelength as SBN.

Rat plasma samples were pretreated by solvent precipitation-reconstitution technique, a simpler and more efficient sample preparation method, compared to the solid phase or liquid–liquid extraction method. To optimize sample preparation procedures, various organic solvents for protein precipitation including acetone, methanol, acetonitrile (ACN), trichloroacetic acid, and their combinations, were examined. Among these, ACN yielded the lowest matrix effect and highest recovery for SBN and DIC with a centrifugation speed of 15,000*g* for an acceptable precipitation period of 5 min.

### 2.2. Method Validation

**Figure 2:**
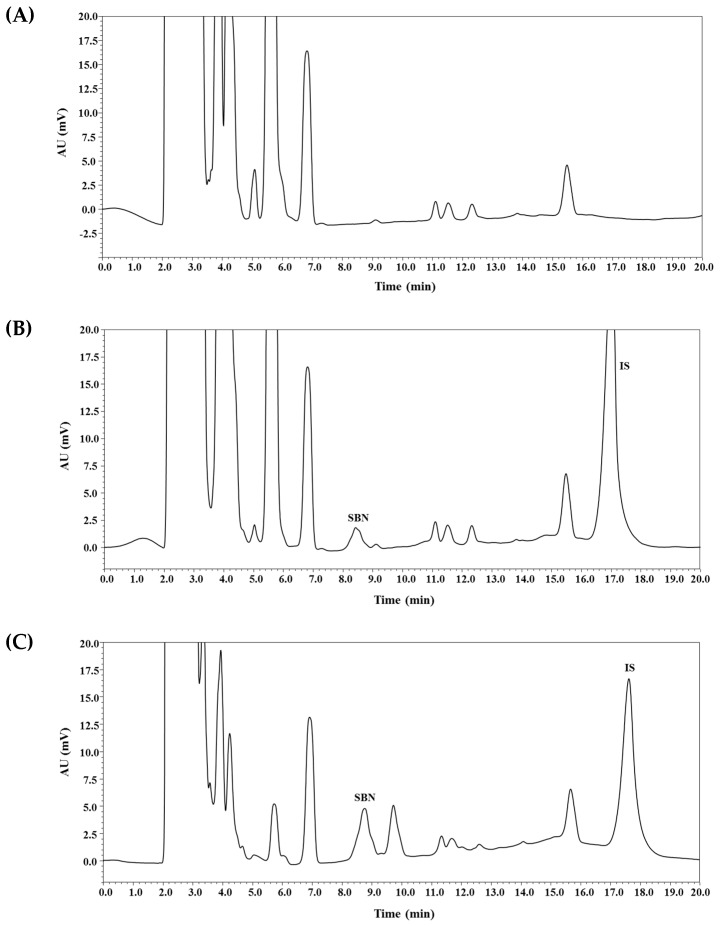
Typical chromatograms of SBN and IS in rat plasma: blank rat plasma (**A**); blank rat plasma spiked with analytes (600 ng/mL, middle quality control (MQC)) and IS (**B**); plasma sample collected at 30 min after oral administration of commercial silymarin product #4 (200 mg/kg as silybin) in rats, where calculated concentrations of SBN was 1055 ng/mL, respectively (**C**).

As shown in Figure 2, SBN peaks were well separated from the peaks of IS and endogenous substances in the blank plasma. These results imply that the bioanalytical method developed herein may provide acceptable selectivity without endogenous interferences occurring at the appearance of SBN and IS peaks. The calibration curves (SBN-to-IS peak area ratio versus SBN-to-IS concentration ratio) for SBN were observed to be linear from 50 to 5000 ng/mL. A representative equation for the calibration curves is as follows: *y* = 0.2224 × *x* + 0.0001, where *y* indicates the ratio of SBN peak area to that of IS, and *x* indicates the ratio of nominal concentration of SBN to that of IS. The correlation coefficients (*r*^2^) were more than 0.999, indicating an acceptable linearity of our method. The intra- and inter-day accuracy and precision were determined for SBN at the LLOQ (50 ng/mL) and three quality control (QC) levels (3000 ng/mL (high; HQC), 600 ng/mL (middle; MQC), 150 ng/mL (low; LQC)), as shown in Table 1. The precision of the method was determined to be 8.8% or less, and its accuracy ranged from 96.6% to 111.2%. These values are within the acceptable range, indicating that the present method is reproducible, accurate, and precise. Notably, our present method with a simple protein deproteinization procedure achieved an equivalent LLOQ (50 ng/mL) in a previous study involving liquid–liquid extraction [4].

The recovery and matrix effects were determined for SBN at the QC levels and for IS at 1000 ng/mL (Table 2). The recovery of SBN was 92.3%–100.1% with CV values of <4.4%. The mean matrix effect was 91.4%–98.8% with CV values of <5.4%. The SBN stability was measured under various conditions relevant to the present method. The bench-top, freeze-thaw, autosampler, and long-term stabilities were measured for SBN at the levels tested. We observed the bias in the concentration to be within ±15% of the corresponding nominal value; the remaining fraction of SBN was 88.6%–113.3% with CV values of <5.0% (Table 3). Our results show that the sample preparation procedures used herein could offer acceptable matrix effect with good extraction recovery, and that SBN remains stable under various storage and handling conditions relevant to this bioanalytical method.

### 2.3. Application to a Comparative Pharmacokinetic Study in Rats

**Figure 3:**
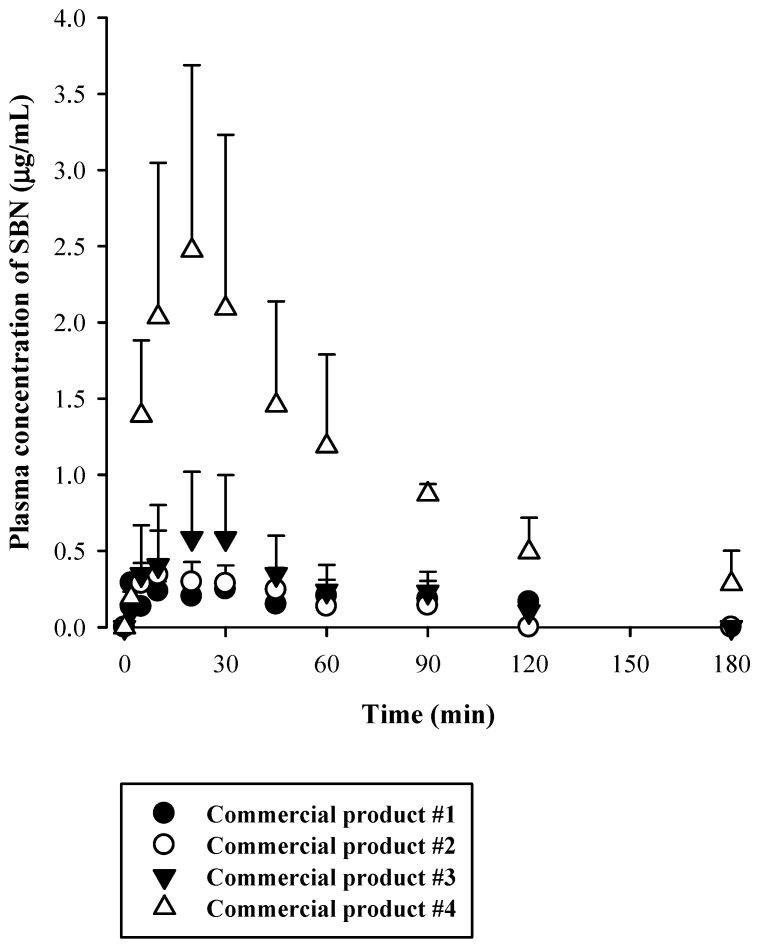
Plasma concentration versus time profiles of SBN following oral administration of four different commercial silymarin products in rats (n = 5).

Rats were administered oral silymarin products at 200 mg/kg as SBN. Following this, plasma concentrations versus time profiles of SBN were assessed (Figure 3). Their relevant pharmacokinetic parameters are listed in Table 4. The oral SBN dose was selected based on previous preclinical pharmacokinetic studies on SBN [4,5,8,9]. We identified linear terminal phases of plasma concentration profiles obtained from oral studies. After the oral administration of the four silymarin products, plasma SBN levels reached respective peaks (C_max_) at 2–30 min, which was rather consistent with previously reported rat data (11 ± 1.8 min) [5]. As shown in Table 4, the C_max_, AUC_inf_, and AUC_last_ of SBN after oral administration of commercial product #4 were significantly higher than those after administration of the other commercial silymarin products #1–3 as follows: the AUC_inf_ after administration of product #4 was 6.3-, 8.3-, and 4.4-fold higher; the AUC_last_ after administration of product #4 was 8.9-, 11.8-, and 6.2-fold higher; and the C_max_ after administration of product #4 was 12.5-, 6.9-, and 4.2-fold higher than those after administration of products #1–3, respectively. These results clearly indicated that the commercial silymarin products exhibited fast oral absorption of SBN and that the bioavailability of SBN was higher with product #4 than with the other three products.

## 3. Materials and Methods

### 3.1. Materials

SBN (purity ≥95.0%), potassium phosphate monobasic/dibasic, and dimethyl sulfoxide (DMSO) were obtained from Sigma-Aldrich Co. (St. Louis, MO, USA). Diclofenac (purity ≥98.0%) was obtained from Tokyo Chemical Industry Co. (Tokyo, Japan). HPLC-grade acetonitrile (ACN) was obtained from Honeywell, Inc. (Muskegon, MA, USA). The silymarin products from four different manufacturers, indicated as ′Commercial product #1–4′, were evaluated. The names of the manufacturers were not disclosed for privacy reasons.

### 3.2. Calibration Standards and QC Samples

Stocks of SBN and IS were prepared in DMSO at 1 mg/mL. The stock solution of SBN was diluted serially using methanol to make working solutions with concentrations 5–500 μg/mL. The working solution of IS (100 μg/mL) was prepared in ACN. The calibration standard samples were made by spiking the blank rat plasma with each corresponding working solution, thereby yielding final concentrations of 50, 100, 200, 500, 1000, 2000, and 5000 ng/mL. The QC samples were prepared with new stock solutions of SBN using the same procedure adopted in preparing calibration standards.

### 3.3. Chromatographic Conditions

A Shimadzu HPLC-UV system with a pump (LC-20AT), an autosampler (SIL-20AC), a column oven (CTO-20A), and an ultraviolet detector (SPD-20A) was used in this study (Shimadzu Co., Kyoto, Japan). A Kinetex C18 column (250 × 4.6 mm, 5 μm, 100 Å; Phenomenex, Torrance, CA, USA) protected by a C18 guard column (SecurityGuard HPLC Cartridge System, Phenomenex) was used for chromatographic separation at 40 °C. The mobile phase for the HPLC system consisted of phosphate buffer (pH 5.0; 10 mM) (solvent A) and ACN (solvent B) at a flow rate of 1 mL/min. The gradient elution protocol was as follows (solvent A/solvent B (*v*/*v*)): ramped from 71:29 (*v*/*v*) to 59:41 (*v*/*v*) during 10 min; back to 71:29 (*v*/*v*) for 10 min. The wavelength for SBN and IS was set as 220 nm. The total run time and injection volume were 20 min and 20 μL, respectively. All solvents used for sample preparation and HPLC analysis were degassed by sonication under vacuum and in-line electronic vacuum degasser modules.

### 3.4. Method Validation

The bioanalytical method for determining SBN in biological samples was validated as per the US FDA guidelines [16]. The selectivity, linearity, precision, accuracy, recovery, matrix effect, bench–top, freeze–thaw, post–preparative (autosampler), and long-term stabilities were evaluated as previously described [17,18].

### 3.5. Pharmacokinetic Study in Rats

Male, 9-week-old Sprague–Dawley rats (approximately 300 g) were purchased from Samtako Bio Korea Co. (Gyeonggi-do, South Korea). Animal study protocols used in this study were approved by the Pusan National University-Institutional Animal Care and Use Committee (approval number: PNU-2018-1848). Rats were fasted during 12 h and anesthetized by intramuscular injection of zoletil at 20 mg/kg. Their femoral arteries were cannulated with a polyethylene tube (Clay Adams) 4 h before drug administration. The rats were randomly divided into four treatment groups (n = 5 per group) receiving four different commercial silymarin products. All the products tested are currently marketed in soft gelatin capsules. Their undiluted liquid contents, with no further formulation, were administered orally to rats at a single dose of 200 mg/kg as silybin. Approximately 300 μL blood was collected through the femoral artery at 0, 2, 5, 10, 20, 30, 45, 60, 90, 120, and 180 min after the oral dosing. Following centrifugation of the blood sample at 2000*g* at 4 °C for 10 min, a 120 μL aliquot of the plasma was stored at −20 °C until HPLC analysis.

### 3.6. Sample Pretreatment

A plasma sample (120 μL) was deproteinized with 400 μL of ice-cold ACN (IS dissolved at 1000 ng/mL). After vortexing during 5 min and centrifugation for 5 min at 15,000*g*, 400 μL supernatant was transferred to a 1.7 mL microcentrifuge tube and dried under a gentle nitrogen gas stream. The resultant residue was reconstituted with 40 μL mobile phase, and a 20 μL aliquot was analyzed by the HPLC system.

### 3.7. Data Analysis

The analytical data acquisition and processing were conducted using the LC Solution Software (Version 1.25; Shimadzu Co.). All chromatograms were analyzed using the IS. Peak area ratios of SBN to IS were used for calculations (least squares regression, weighting factor of 1/*x*, *x* = concentration). For pharmacokinetic analysis, non-compartmental analysis (WinNonlin, version 3.1, NCA200 and 201; Certara USA Inc., Princeton, NJ, USA) was used to estimate the following pharmacokinetic parameters: total area under the plasma concentration versus time curve from time zero to the last sampling time (AUC_last_) and to time infinity (AUC_inf_). The peak plasma concentration (C_max_) and time to reach C_max_ (T_max_) were directly read from observed data.

### 3.8. Statistical Analysis

Statistical analysis was conducted using *t*-test for comparing unpaired two means or Tukey′s honestly significant difference (HSD) test with posteriori analysis of variance (ANOVA) for comparing unpaired three means. A p-value < 0.05 indicated statistical significance. Unless indicated otherwise, all data are expressed as mean ± standard deviation and rounded to one decimal place, except T_max_ values expressed as median (range) and as an integer number.

## 4. Conclusions

A simple and efficient HPLC–UV method was successfully developed and validated for the quantitative determination of SBN in rat plasma. The developed method offers several advantages including simplicity of sample preparation procedures, good recovery, negligible matrix effect, and acceptable sensitivity comparable to the previously reported HPLC method which employs more complex sample pretreatment procedure. The comparative pharmacokinetic study on commercial silymarin products revealed that product #4 significantly enhanced the relative oral bioavailability of SBN compared to product #1–3. Therefore, the bioanalytical method proposed herein could serve as a promising alternative for preclinical pharmacokinetic studies on silymarin products and, by extension, clinical use after partial modification and validation.

## Figures and Tables

**Table 1:** Intra- and inter-day precision and accuracy of SBN in rat plasma (n = 5).

Nominal Concentration (ng/mL)	Precision (%)		Accuracy (%)	
	Intra-Day	Inter-Day	Intra-Day	Inter-Day
LLOQ (50)	4.3	4.3	106.7	103.2
LQC (150)	1.4	7.1	104.1	100.6
MQC (600)	2.3	8.8	107.4	96.6
HQC (3000)	3.1	8.5	111.2	99.3

**Table 2:** Recovery and matrix effect of SBN and IS in rat plasma (n = 5).

Nominal Concentration (ng/mL)	Recovery (%)	Matrix Effect (%)
LLOQ (50)	100.1 ± 4.5	95.0 ± 5.1
LQC (150)	98.6 ± 2.1	92.9 ± 4.6
MQC (600)	93.6 ± 1.5	91.4 ± 1.8
HQC (3000)	92.3 ± 3.9	98.0 ± 1.4
IS (Diclofenac, 1000)	93.3 ± 3.1	98.8 ± 1.6

**Table 3:** Stability (%) of SBN in rat plasma (n = 5).

Nominal Concentration (ng/mL)	Bench–Top ^a^	Autosampler ^b^	Freeze–Thaw ^c^	Long–Term ^d^
LLOQ (50)	103.7 ± 5.2	91.8 ± 3.5	95.1 ± 1.7	92.3 ± 4.4
LQC (150)	100.3 ± 3.6	88.6 ± 1.6	98.4 ± 3.2	93.6 ± 3.4
MQC (600)	101.5 ± 2.3	107.1 ± 3.7	94.7 ± 1.6	100.5 ± 2.0
HQC (3000)	100.7 ± 0.9	98.1 ± 3.1	113.3 ± 1.6	98.6 ± 0.3

^a^ Room temperature during 3 h. ^b^ 25 °C during 24 h in the autosampler. ^c^ Three freezing and thawing cycles. ^d^ −20 °C during 14 days.

**Table 4:** Pharmacokinetic parameters of SBN following oral administration of four different commercial silymarin products in rats (n = 5).

Parameter	AUC_inf_ (μg‧min/mL)	AUC_last_ (μg‧min/mL)	C_max_ (μg/mL)	T_max_ (min)
Commercial product #1	35.6 ± 16.2	23.2 ± 13.6	0.250 ± 0.056	30 (2–30)
Commercial product #2	26.9 ± 11.2	17.6 ± 11.0	0.455 ± 0.277	10 (5–30)
Commercial product #3	50.6 ± 24.9	33.2 ± 18.6	0.744 ± 0.331	10 (5–20)
Commercial product #4	224.5 ± 37.4 *	207.0 ± 28.0 *	3.13 ± 0.49 *	20 (10–20)

* Significantly different from the other groups (p < 0.05).
